# *Swiss cheese* Is Essential for Maintaining Spermatogenesis and the Proper Functioning of Biological Barriers in *Drosophila*

**DOI:** 10.3390/ijms27125486

**Published:** 2026-06-17

**Authors:** Elena V. Ryabova, Ekaterina A. Ivanova, Artem E. Komissarov, Elena U. Bolobolova, Natalia V. Dorogova, Elizaveta E. Slepneva, Evgenia M. Latypova, Irina V. Ogneva, Svetlana V. Sarantseva

**Affiliations:** 1 Petersburg Nuclear Physics Institute Named by B.P. Konstantinov of National Research Centre «Kurchatov Institute», 188300 Gatchina, Russia; ryabova_ev@pnpi.nrcki.ru (E.V.R.); ivanova_ea@pnpi.nrcki.ru (E.A.I.); komissarov_ae@pnpi.nrcki.ru (A.E.K.); slepneva_ee@pnpi.nrcki.ru (E.E.S.); latypova_em@pnpi.nrcki.ru (E.M.L.); 2 Institute of Cytology and Genetics, Siberian Branch of Russian Academy of Sciences (ICG SB RAS), 630090 Novosibirsk, Russia; elbol@bionet.nsc.ru (E.U.B.); dorogova@bionet.nsc.ru (N.V.D.); 3 State Scientific Center of the Russian Federation Institute of Biomedical Problems, Russian Academy of Science, 123007 Moscow, Russia; iogneva@yandex.ru

**Keywords:** *Swiss cheese*, *PNPLA6*, *Drosophila melanogaster*, spermatogenesis, septate junctions, somatic permeability barrier

## Abstract

Functional changes in *PNPLA6* (Patatin-like phospholipase domain-containing protein 6), caused by gene mutations or inhibition by organophosphates, affect the levels of various phospholipids. In humans, this leads to organophosphorus compound-induced delayed neurotoxicity syndrome (OPIDN) and a number of rare diseases. In this study, we analyze the role of the *Swiss cheese* gene (*sws*), an ortholog of *PNPLA6*, in spermatogenesis in *Drosophila melanogaster*. We report that the *sws*^1^ mutation affects membrane remodeling during spermatid individualization, as well as spermatid coiling during the late stages of spermatogenesis. In addition, the *sws*^1^ mutation leads to changes in the transcriptome in the testes of flies. We also demonstrate that *sws* is required for the proper functioning of important biological barriers in *Drosophila*.

## 1. Introduction

Patatin-like phospholipase domain-containing proteins (PNPLAs) play a crucial role in cell membrane renewal, the generation of signaling molecules, and lipid metabolism [1,2,3]. PNPLAs hydrolyze lipid substrates such as neutral lipids or glycerophospholipids [4]. A link has been shown between mutations in the *PNPLA* family genes or disruption of the catalytic activity of the proteins they encode and the development of various diseases [5,6,7].

*PNPLA6* (historically known as neurotoxic esterase (*NTE*)) has been identified as a target of organophosphorus compounds (OPs) that cause organophosphorus compound-induced delayed neurotoxicity (OPIDN) syndrome characterized by paralysis of the lower limbs due to long axon degeneration [4]. Mutations in the *PNPLA6* gene are also responsible for autosomal recessive spastic hereditary paraplegia 39 (SPG39) [6] and other rare neurological diseases such as ataxia with spasticity or pure cerebellar ataxia [8,9,10], Gordon Holmes syndrome, Boucher–Neuheuser syndrome, Lawrence–Moon syndrome, Oliver–McFarlane syndrome and Leber congenital amaurosis [11,12,13,14].

Orthologs of the *PNPLA6* gene have been found in various organisms: nematodes, yeast, bacteria, *Drosophila*, and vertebrates [15]. All encode a phospholipase B that deacylates (lyso)phosphatidylcholine to glycerophosphocholine and one/two molecules of free fatty acids [1,16,17,18]. *PNPLA6* and its orthologs are widely expressed in the nervous system; expression has also been detected in testes [19,20], kidneys [21], and liver cells [19].

The ortholog of the human *PNPLA6* gene in *Drosophila melanogaster* is the *Swiss cheese* gene (*sws*), the function of which is important for neuronal viability [16,22], as well as the normal structure and function of glial cells, particularly subperineural glia, which are part of the *Drosophila* blood–brain barrier (BBB). This gene was identified in 1979 in the laboratory of M. Heisenberg during the analysis of mutants with anatomical brain defects resulting from exposure to the chemical mutagen ethylmethanesulfonate. Several mutations in the functional esterase domain and a nonsense mutation leading to the loss of one third of the protein (*sws*^1^, C7963853A, Ser375*) have been identified in the *sws* gene [16,22,23,24].

Previously, we first described the normal expression pattern of *sws*, identifying it in the male reproductive system, and demonstrated that this gene is critical for male fertility [25]. The mutant males had reduced courtship activity, their fertility significantly decreased with age, and sperm motility was reduced [26]. However, the reason for this phenotype remains unclear. Therefore, we hypothesized that *sws* might play a role in spermatogenesis. Spermatogenesis of *Drosophila* begins at the apical tip of the testes, where a stem cell niche is located, which includes hub cells, somatic cyst stem cells (CySCs), and germline stem cells (GSCs). These cells are in close contact with each other. The hub acts as a signaling center for both types of stem cells, providing their trophic support. Each of the eight GSCs divides asymmetrically to form one daughter cell, which remains in the niche and supports a pool of stem cells, and a gonialblast, which moves away from the hub and begins differentiation. CySCs also divide for self-renewal and cyst cell formation. Two CySCs encapsulate the gonialblast and differentiate with it [26,27]. The formed gonialblasts undergo four stages of mitotic division with the formation of cysts from 16 spermatogonia [27,28]. Next, they undergo meiotic division, and cysts of 64 haploid spermatids are formed, which then undergo a number of morphological changes necessary for the development of spermatozoa: the elongation of spermatid tails and nucleus shape modification [26]. In the end, the individualization of spermatids occurs when they are encapsulated in their own membranes. This requires the formation of an individualization complex (IC) consisting of 64 F-actin cones. Their main function is to carry out the movement of the individualization complex and membrane remodeling [29]. At the beginning of individualization, cones form around each of the nuclei in the mature cyst and then synchronously move along the tails towards the apical tip of the testis to form a cyst bladder, and each spermatozoa becomes enclosed in its own plasma membrane [30]. After individualization, each group of mature spermatozoa folds into a spiral, after which they are released into the lumen of the testis and transferred to the seminal vesicle for storage [26,31].

In this study, we studied spermatogenesis in the *sws*^1^ mutant and identified that *sws* is essential for the individualization of the spermatid and the maintenance of barrier functions in the body.

## 2. Results

### 2.1. sws^1^ Mutant Testes Showed Normal Early Spermatogenesis and Elongation

**Figure 1:**
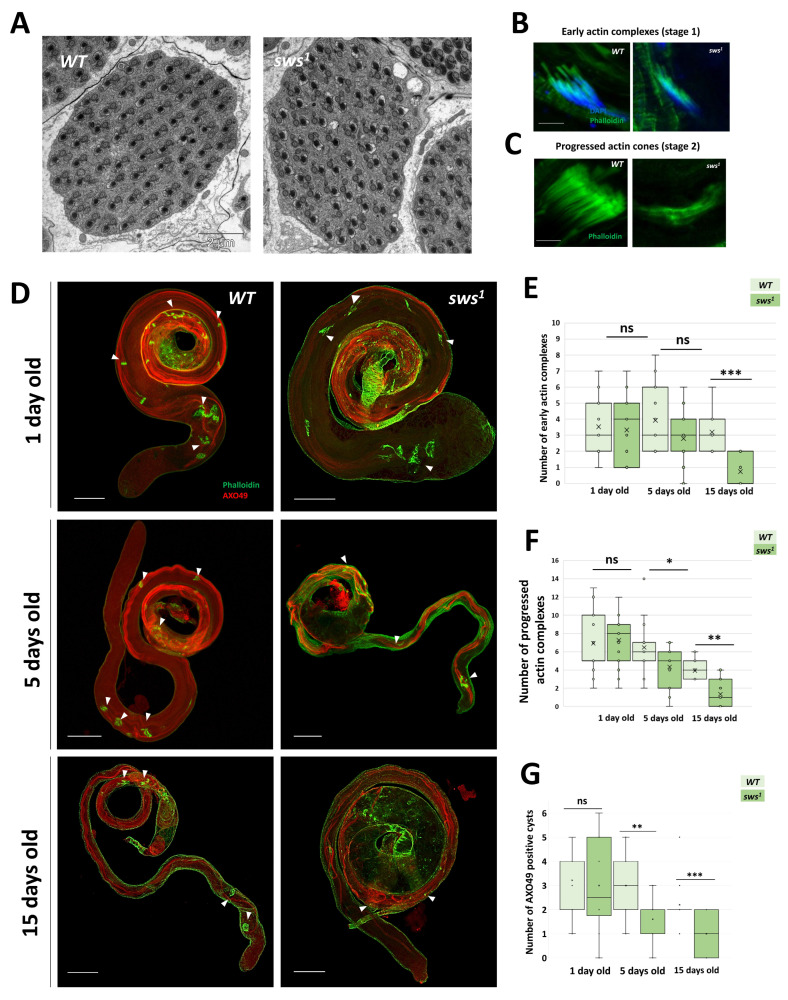
Individualization of spermatids in *Drosophila*. (**A**) Transmission electron micrograms of the elongating cysts of the WT and *sws*^1^ flies. Scale bar: 2 µm. (**B**) Early individualization complexes. Blue—Dapi. Green—phalloidin iFluor488. Scale bar: 10 µm. (**C**) Progressed individualization complexes. Green—phalloidin iFluor488. Scale bar: 10 µm. (**D**) Individualization of the spermatid in the WT and *sws*^1^ mutant. The white arrowheads show actin complexes at different stages of individualization. Red—AXO49. Green—phalloidin iFluor488. Scale bar: 100 µm. (**E**) Number of early individualization complexes in 1-, 5-, and 15-day-old WT and *sws*^1^ mutants. Box plot, Student’s *t*-test. ns—no significant difference (*p* > 0.05); ***—*p* ≤ 0.001. *N* = 15. (**F**) Number of progressed actin complexes in 1-, 5-, and 15-day-old WT and *sws*^1^ mutants. Box plot, Student’s *t*-test. ns—no significant difference (*p* > 0.05); *—*p* ≤ 0.05; **—*p* ≤ 0.01. *N* = 15. (**G**) Number of AXO49-positive cysts in 1-, 5-, and 15-day-old WT and *sws*^1^ mutants. Box plot, Mann–Whitney U test. ns—no significant difference (*p* > 0.05); **—*p* ≤ 0.01; ***—*p* ≤ 0.001. *N* = 15.

To find out the reason for low fertility in males, we performed a detailed cytological analysis of spermatogenesis in *sws*^1^ mutants by transmission electron microscopy (TEM). The results of the analysis showed that spermatogenesis generally progresses to the stage of individualization of spermatids and that we did not detect phenotypic defects in the morphology, dynamics, and remodeling of intracellular structures. In the late stage of elongation, we observed a characteristic normal spermatid morphology in both the wild type (WT) and the *sws*^1^ mutant. In TEM cross sections, each spermatid contains mitochondrial derivatives and axonemal microtubules. The major derivative is filled with paracrystalline material, and the minor derivative has a smaller volume. Mitochondria in combination with axonemes form an axial complex, which at this stage is surrounded by a large amount of cytoplasmic material (Figure 1A). Normally, this cytoplasmic material is almost completely removed during the next stage of individualization.

### 2.2. Spermatid Individualization Is Disrupted in sws^1^ Testes

**Figure 2:**
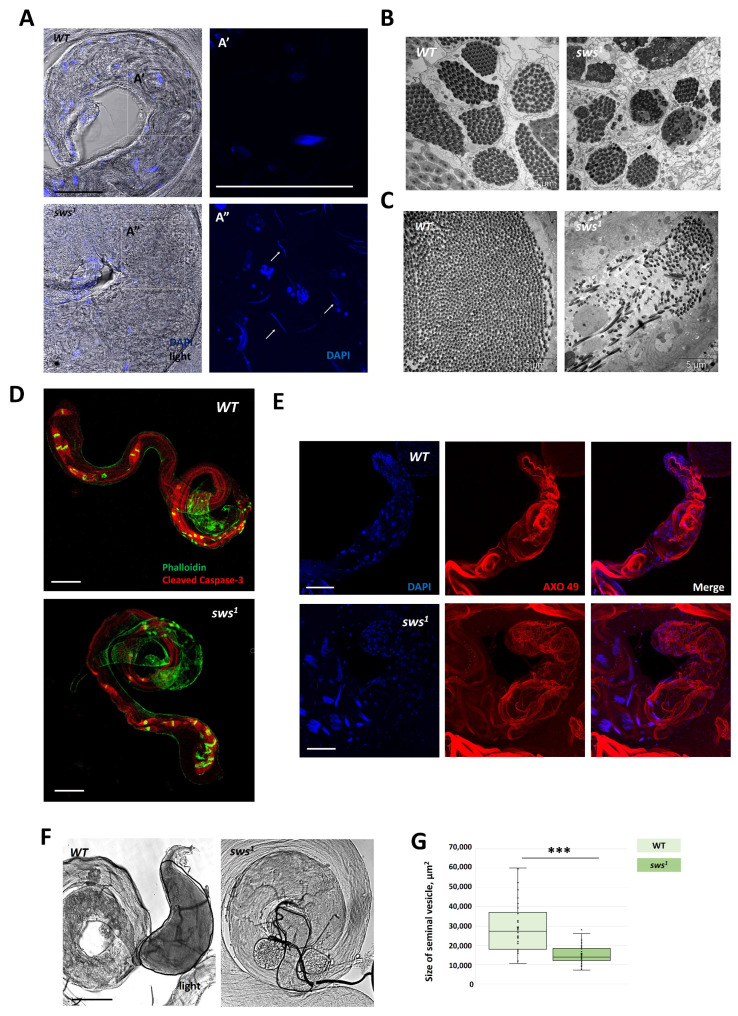
The dysfunction of the *sws* gene in cyst cells is affected during the late stages of spermatogenesis. (**A**) TE of the testes in the 5-day-old WT and *sws*^1^ flies. Light microscopy. (**A′**,**A″**) Single nuclei in TE (indicated by arrows). Blue—Dapi. Scale bar: 50 µm. (**B**) Transmission electron micrograms of 64-spermatid cysts in 5-day-old WT and *sws*^1^ mutants. Scale bar: 5 µm. (**C**) Transmission electron micrograms of the seminal vesicle incision in 5-day-old WT and *sws*^1^ flies. Scale bar: 5 µm. (**D**) Non-apoptotic caspase cascade in cystic bulges. Red—Cleaved Caspase-3. Green—phalloidin iFluor488. Scale bar: 100 µm. (**E**) Coiled tails of mature sperm groups in the TE. Blue—Dapi; red—AXO49. Scale bar: 50 µm. (**F**) Light microscopy images of the seminal vesicles (black lines) in 5-day-old WT and *sws*^1^ flies. Scale bar: 100 µm. (**G**) Size of the seminal vesicles in 5-day-old WT and *sws*^1^ flies (µm^2^). Box plot, Mann–Whitney U test. ***—*p* ≤ 0.001. *N* = 30.

Individualization of spermatids begins with the formation of ICs around nuclei in mature cysts of early spermatids, in particular composed of F-actin [32]. We visualized ICs using the fluorescent dye phalloidin conjugated to iFluor488 (Figure 1B,C) and polyglycylated tubulin as a marker of developed elongated spermatids and, consequently, elongated cysts using AXO 49 antibodies (Figure 1D and Figure S2). Actin complexes are present in the WT and in the *sws*^1^ mutant in 1-day-old flies, but the number of ICs, as well as the number of actin complexes moving along the tail of the spermatids and the number of AXO-positive cysts, decreases in the *sws*^1^ mutant at 5 days old, and they are almost absent at 15 days old (Figure 1E,G). In the late stages of spermatogenesis in *sws*^1^, the nuclei of the spermatids have a normal needle shape, but it should be noted that no nuclear bundles were detected (Figure 2A, shown by the arrows).

The spermatid individualization process also involves non-apoptotic activation of caspases to dispose of the bulk of the cytoplasm and organelles that accumulate as actin complexes move along the spermatid tails. Using antibodies specific for activated caspase 3, we detected that the non-apoptotic caspase cascade was active in the formation of cystic bulges and waste bags in sws1 mutants and did not differ from that of the WT (Figure 2D and Figure S2).

In the WT, individualization leads to separation of spermatids; as a result, part of the cytoplasm is removed and each spermatid is surrounded by a plasma membrane. Spermatids are tightly packed and highly ordered. In *sws*^1^ mutant cysts, we observed a lack of spermatid individualization. Spermatids do not separate and remain in the common cytoplasm. A significant proportion of the cysts examined contained separate, individualized spermatids and a cluster of unindividualized ones (Figure S1). Subsequently, these clusters lose their internal structure and become denser and darker (Figure 2B). Probably, this is how their degradation manifests itself.

In the final stages of spermatogenesis, each group of mature sperm coils in the terminal epithelium (TE) and is released into the testicular lumen before migrating from the TE to the seminal vesicle, where they prepare for ejaculation [26,27]. Visualization of mature spermatids with AXO49 antibodies revealed that the *sws*^1^ mutant, unlike the WT, exhibits disorganization of the mature spermatid tails in the TE, appearing as tangled tails (Figure 2E).

As a result of abnormal individualization, a low number of mature sperm are formed (Figure 2C). As a result of the processes that take place, a decrease in the size of the seminal vesicles is observed in the *sws*^1^ mutant (Figure 2F,G).

**Figure 3:**
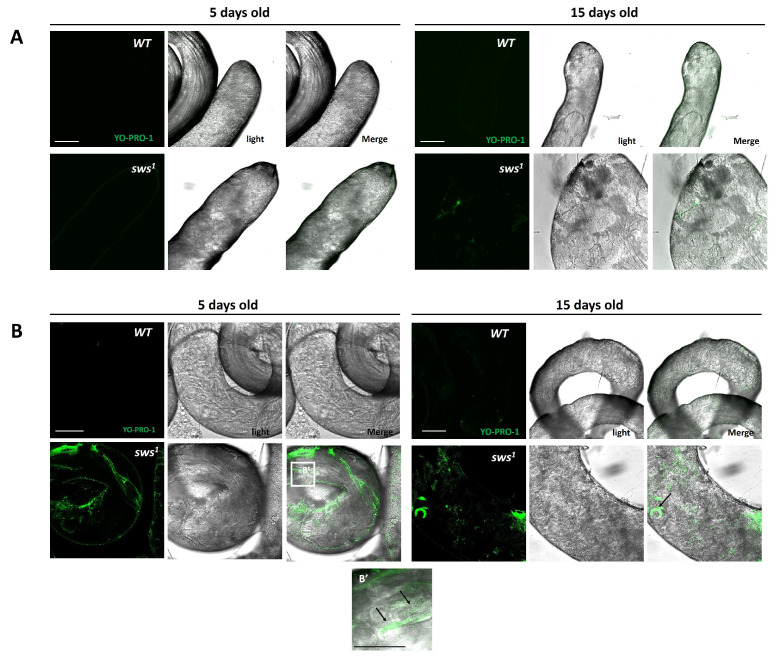
Analysis of cyst cell apoptosis. (**A**) Apical tips of testes of different ages. (**B**) TE of testes of different ages. (**B′**) Location of YO PRO-1 in spermatids (showed by black arrows). Green—YO PRO-1. Light microscopy. Scale bar: 50 µm.

To determine whether mature spermatids in TE are viable, we stained the testes with the fluorescent dye YO PRO-1. YO-PRO-1 is a nuclear marker that does not penetrate the intact plasma membrane of living cells due to its size. However, it binds to the DNA of dying cells due to alterations in the cell plasma membrane during apoptosis [33]. In WT, the dye did not penetrate into the testis cells of flies of different ages (Figure 3). In *sws*^1^, the dye did not penetrate cyst cells only in the early stages of spermatogenesis (Figure 3A). In the post-meiotic stages, the dye accumulates in the cyst cells of the early and late spermatids (Figure 3B).

### 2.3. Gene Expression Profile Is Altered in sws^1^ Testes

**Figure 4:**
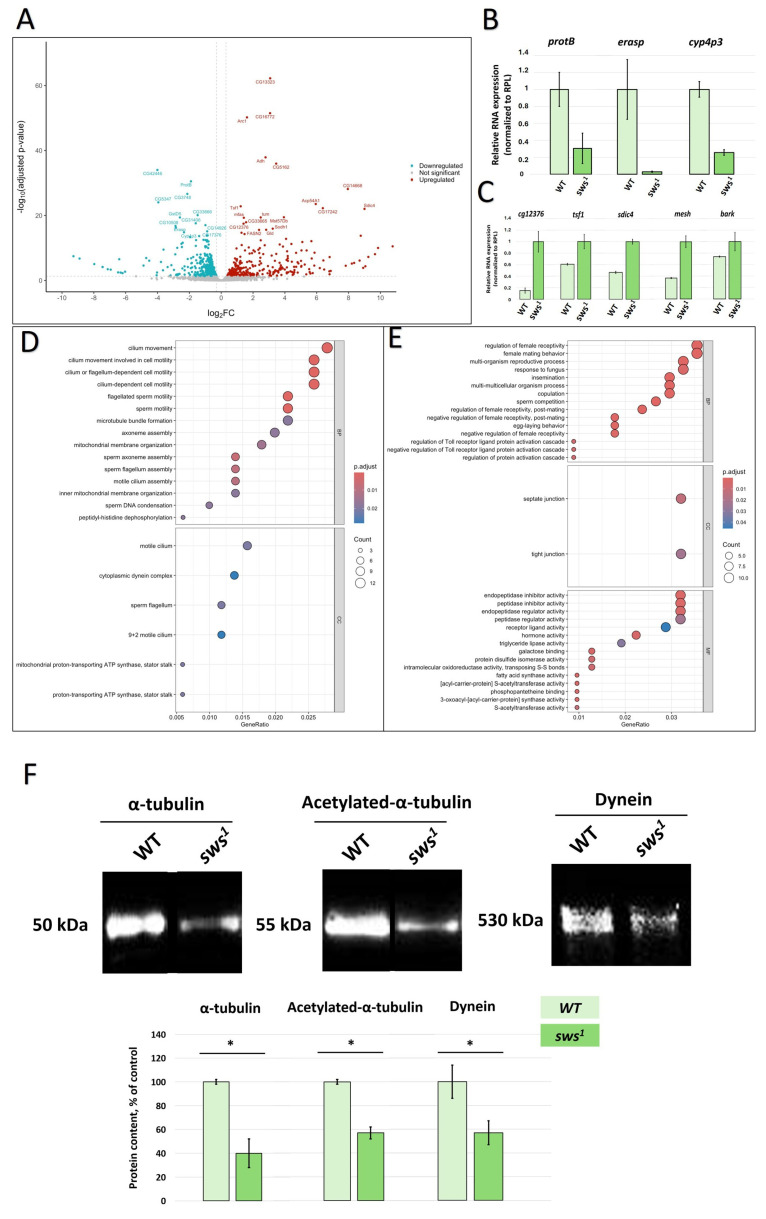
RNA sequencing analysis showing differential gene expression of the WT and the *sws* mutant. (**A**) Volcano plot showing the log10 of the adjusted *p*-value vs. log2 fold change. The dashed vertical lines mark the log2 fold change > |0.3|. The dashed horizontal line indicates the adjusted *p*-value < 0.05. Blue dots represent downregulated genes, and red dots represent upregulated genes. (**B**,**C**) qRT-PCR analysis of differentially expressed genes in the WT and *sws*^1^ testes. (**B**) Downregulated genes (*protB*, *erasp*, and *cyp4p3*). (**C**) Upregulated genes (*cg12376*, *tsf1*, *sdic4*, *mesh*, and *bark*). Data are shown as means ± SEMs. (**D**,**E**) Gene Ontology function and pathway enrichment analysis of downregulated DEGs (**D**) and upregulated DEGs (**E**). The terms for each of the GO analysis categories’ BPs, MFs and CCs are presented. (**F**) Relative content of cytoskeletal proteins, participation in sperm motility: α-tubulin protein (50 kDa), acetylated-α-tubulin protein (55 kDa), dynein protein (530 kDa). Data are shown as means ± SEMs. *—*p* ≤ 0.05. *N* = 60.

To more fully characterize the changes in spermatogenesis that occur with *sws* gene knockout, we compared gene expression profiles in the testes of *sws*^1^ and WT males at 5 days of age using RNA sequencing. We identified 1054 differentially expressed genes (DEGs) in the transcriptome data of WT and *sws*^1^ testes of flies. Among them, 389 DEGs were upregulated and 665 DEGs were downregulated in the *sws*^1^ testes (Figure 4A, Supplementary Material, Table S1), indicating a significant restructuring of physiological processes in *sws*^1^.

Next, to understand the role of genes and their interactions in various biological processes, we performed Gene Ontology (GO) enrichment analysis using. clusterProfiler (Version 4.18.4) GO classifies the characteristics of genes and gene products into three domains: biological processes (BPs), molecular functions (MFs), and cellular components (CCs). DEGs are mainly enriched in the motility of the cilium and flagellum cells, the path of sperm motility and reproductive and mating behavior. The protein products of these genes have various MFs, namely, peptidase inhibitor and regulator activity and receptor and hormone activity. It is interesting that these proteins are localized in the cell mainly in the motile cilium, sperm flagellum, cytoplasmic dynein complex, and septate and tight junctions (Figure 4D,E).

These transcriptome results were confirmed by measurements based on qRT-PCR of several genes with the most altered expression (*protB*, *erasp*, *cyp4p3*, *cg12376*, *tsf1*, and *sdic4*), as well as genes of septate junctions (*bark* and *mesh*) (Figure 4B,C).

Among DEGs with reduced expression, genes controlling sperm motility are represented. Analysis of alpha-tubulin, its acetylated form, and dynein levels, which mediate sperm motility, revealed decreased levels, which explains the reduced sperm motility in mutant *sws*^1^ (Figure 4F) [25].

### 2.4. sws Is Required for the Integrity of the Somatic Permeability Barrier of Mature Spermatids

*sws* has previously been shown to be expressed in post-meiotic stages in cyst cells of early and late spermatids [25]. One of the important roles of these cells is maintaining the somatic permeability barrier. In insects, the somatic permeability barrier is formed by septate junctions, which provide paracellular flow between the apical tips and TE of somatic cells, maintaining cell polarity [34]. Taking into account the results of transcriptome analysis indicating the importance of *sws* in septate junctions, we analyzed the integrity of the somatic permeability barrier in adult flies at 1, 5 and 15 days old by injecting 10 kDa dextran conjugated with Texas red into the abdomens of males.

**Figure 5:**
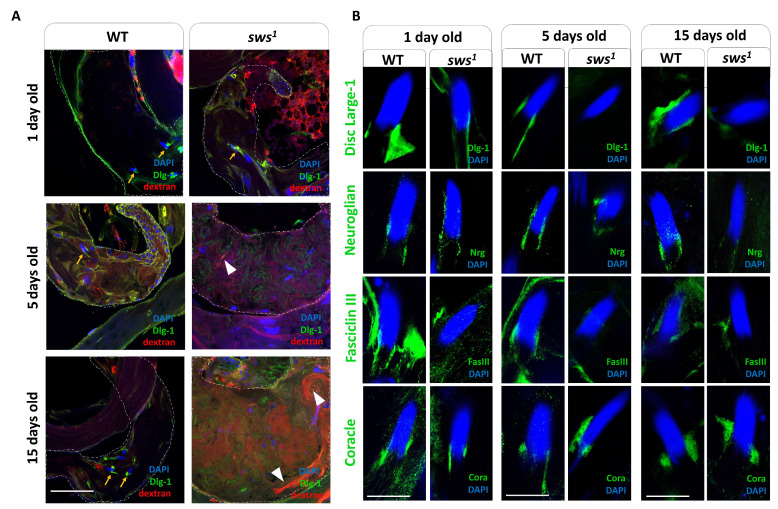
Integrity of the somatic permeability barrier in the late stages of spermatogenesis. (**A**) The somatic permeability barrier becomes leaky in the control and *sws*^1^ mutants of different ages. The white arrowheads show that dextran penetrates mature spermatids. Green—Dlg-1. Red—dextran 10 kDa. Blue—Dapi. The dash line show TE. Yellow arrow shows septate junction. Scale bar: 50 µm. (**B**) Septate junction during the late stage of spermatogenesis in the basal end. The septate junction proteins localize caudally to the compact nuclei bundle of mature spermatids during the late stages, but this localization is absent in *sws*^1^ mutants. Green—Dlg-1, Nrg, FasIII, Cora. Blue—Dapi. Scale bar: 10 µm.

In the early stages of spermatogenesis, neither the *sws*^1^ mutant nor the WT show changes in the integrity of the somatic permeability barrier, which remains impermeable to 10 kDa dextran in flies of different ages (Figure S3). The integrity of the somatic permeability barrier is preserved in the testes of WT flies and at the late stages of spermatogenesis in the TE of the testes (Figure 5A). At the same time, a completely different picture was observed in the testes of *sws*^1^ flies. The barrier was permeable to 10 kDa dextran, indicating a violation of its integrity (Figure 5A and Figure S4).

In *sws*^1^, the septate junction proteins Disks large 1 (Dlg-1), Fasciclin III (FasIII), and Neuroglian (Nrg) do not stain around the caudal end of the compacted nuclei bundle of mature spermatids at the final stages of spermatogenesis (Figure 5B). However, Coracle (Cora) protein was stained in the sws^1^ mutants of different ages. Cora and FasIII belong to the 4.1 family of proteins, which physically link cytoskeletal elements [35,36]. Dlg-1 contains PDZ domains and a guanylate kinase-like domain, suggesting its role in cellular signal transduction. Dlg-1 is required for junctional structure, cell polarity, and proliferation in the *Drosophila* epithelium [37]. Nrg is also a septate junction protein and is believed to be involved in cell adhesion [38].

### 2.5. Overexpression of the sws Gene Against the Background of the sws^1^ Mutation Leads to Restoration of the Individualization of Spermatids and Normal Accumulation of Sperm in the Seminal Vesicles

**Figure 6:**
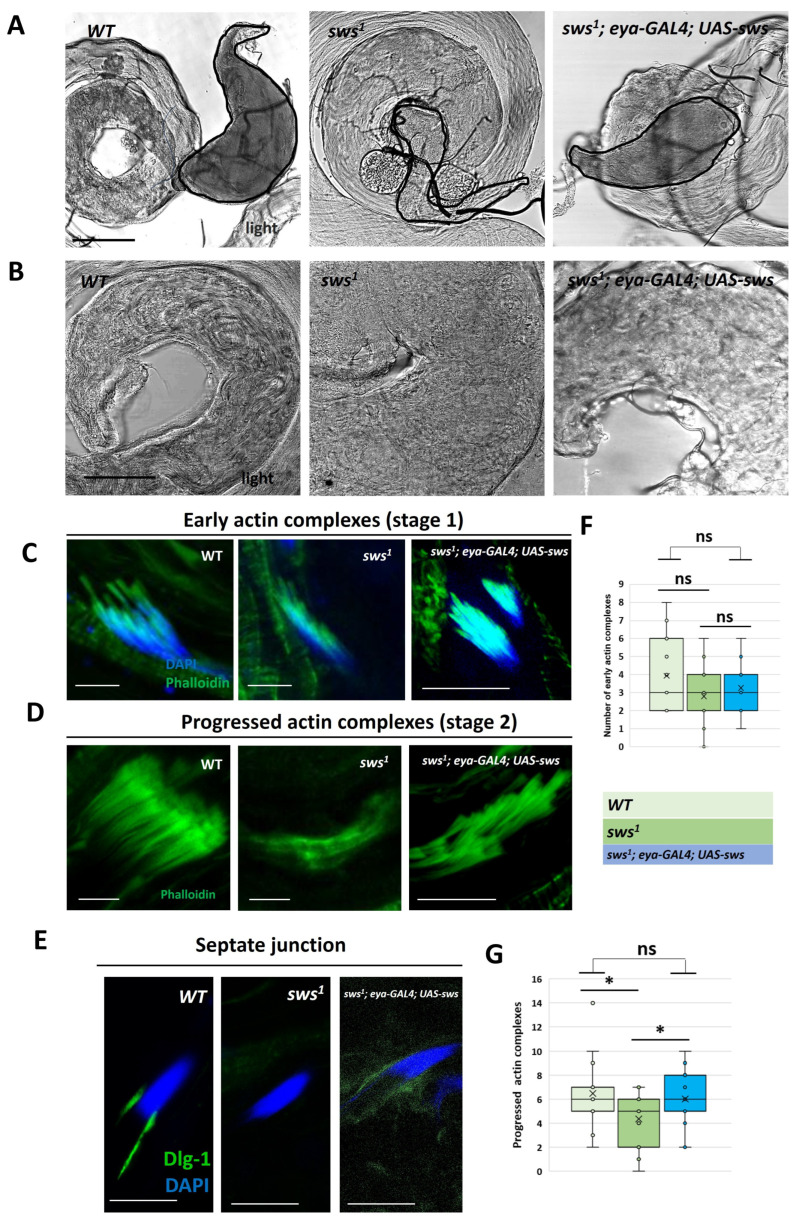
Analysis of individualization of spermatid and sperm accumulation in the seminal vesicle in the *sws*^1^ mutant with late spermatid expression of *sws* (*sws*^1^*;eya-GAL4;UAS-sws*). Age of flies: 5 days old. (**A**) Light microscope images of the seminal vesicles of WT, *sws*^1^ and *sws*^1^*;eya-GAL4;UAS-sws* (black lines). Scale bar: 100 µm. (**B**) Basal side of testis in WT, *sws*^1^ and *sws*^1^*;eya-GAL4;UAS-sws*. Light microscopy. Scale bar: 50 µm. (**C**) Early individualization complexes in WT, *sws*^1^ and *sws*^1^*;eya-GAL4;UAS-sws*. Blue—Dapi. Green—phalloidin iFluor488. Scale bar: 50 µm. (**D**) Progressed individualization complexes in WT, *sws*^1^ and *sws*^1^*;eya-GAL4;UAS-sws*. Green—phalloidin iFluor488. Scale bar: 50 µm. (**E**) The Dlg-1 localizes caudally to the compact nuclei bundle of mature spermatids during the late stages. Blue—DAPI. Green—Dlg-1. Scale bar: 10 µm. (**F**) Histograms of the number of early actin complexes. Box plot, Student’s *t*-test. ns—no significant difference (*p* > 0.05). *N* = 15. (**G**) The histograms of the number of progressed actin complexes. Box plot, Student’s *t*-test. ns—no significant difference (*p* > 0.05); *—*p* ≤ 0.05. *N* = 15.

To confirm that the observed impairments are caused by *sws* knockout, we per-formed a rescue experiment. For this, *sws* overexpression was carried out in spermatids using the *eyaA3-GAL4* driver in an *sws*^1^ background. The size of the seminal vesicle (Figure 6A), the coiling of the spermatids in the TE (Figure 6B), and the individualization of the spermatid (Figure 6C,D,G) were restored in 5-day-old males of the *sws*^1^*;eya-GAL4;UAS-sws* genotype. Furthermore, in the late stages of spermatogenesis, Dlg-1 was located caudally to the bundle of nuclei, forming septate junctions of mature spermatids (Figure 6E).

### 2.6. The sws Gene Is Essential for the Functional Maintenance of Internal Barriers in Drosophila

**Figure 7:**
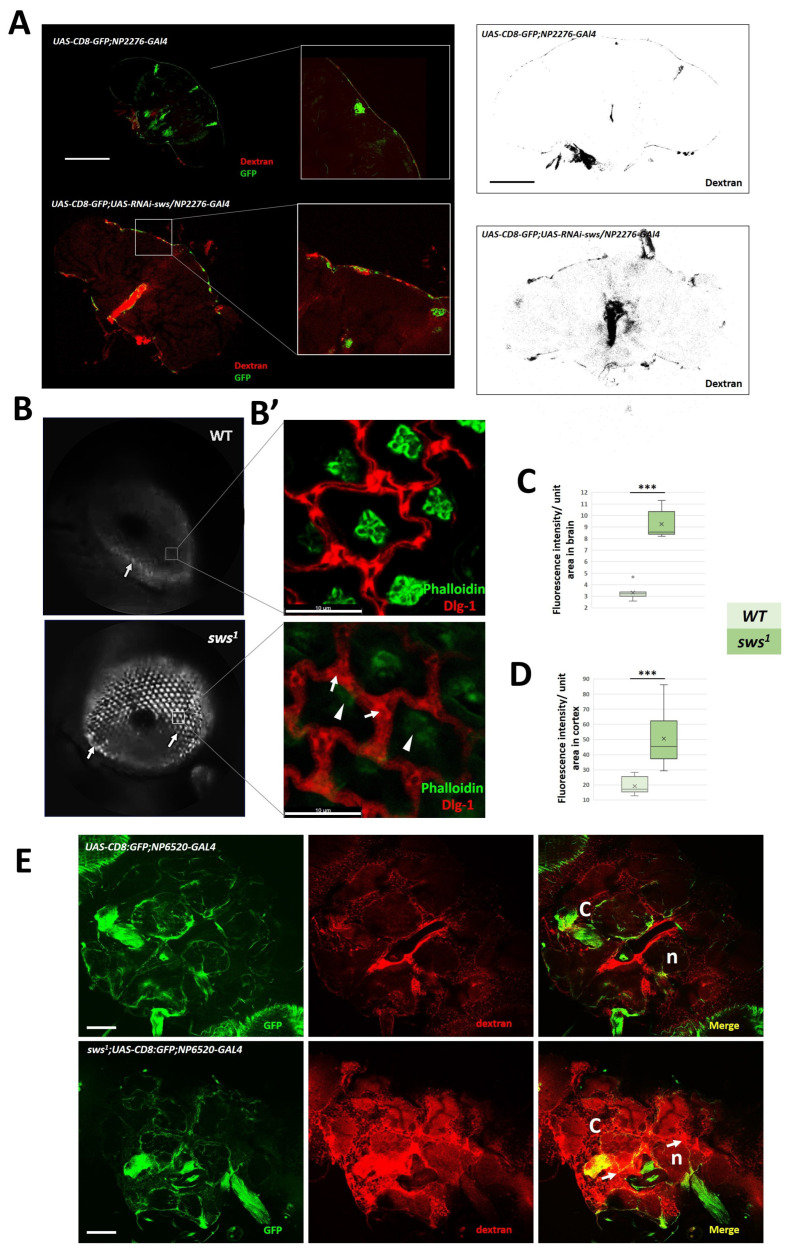
Analysis of barriers in the nervous system of *Drosophila melanogaster.* (**A**) Analysis of BBB. In WT, dextran molecules are concentrated on the surface of the brain and do not pass inside. In the *sws*^1^ mutant, dextran passes through the BBB and is localized inside the brain. Green—CD8-GFP in subperineural glia. Red—Dextran 10 kDa. Scale bar: 100 µm. (**B**) Analysis of blood–eye barrier function. In the WT, dextran is localized on the retina (shown by arrows). The *sws*^1^ mutant allows dye into the retina and demonstrates loss of the hemolymph exclusion line (shown by arrows). (**B′**) Morphology of an adult retina. Green—phalloidin iFluor488 (lamina). Red—Dlg-1 (lateral membrane). In the *sws*^1^ mutant, in the lateral membrane, structures are not clearly visualized (shown by arrowheads) and the structure of the lamina is broken. Scale bar: 10 µm. (**C**) Quantification of dye diffusion in the brain of the genotypes *UAS-CD8-GFP;NP2276-GAL4* and *UAS-CD8-GFP;NP2276-GAL4/UAS-RNAi-sws*. *N* = 7, box plot, Student’s *t*-test; ***—*p* ≤ 0.001. (**D**) Quantification of dye diffusion in the cortex of the genotypes *UAS-CD8-GFP;NP2276-GAL4* and *UAS-CD8-GFP;NP2276-GAL4/UAS-RNAi-sws. N* = 7, box plot, Student’s *t*-test; ***—*p* ≤ 0.001. (**E**) Analysis of ensheathing glia function. n—neuropil; c—cortex. The distribution of the dye in the cortex is shown by arrows. Green—CD8-GFP in the ensheathing glia. Red—dextran 10 kDa. Scale bar: 50 µm.

In addition to the somatic permeability barrier, *Drosophila*, as a multicellular organism, has a number of internal physiological barriers whose main function is to protect the body from pathogenic effects and regulate metabolism. Taking into account our results for *sws* expression, we analyzed a number of internal barriers in the body of *Drosophila* when it is destroyed. It has previously been shown that *sws* dysfunction leads to subperineural glia death [23,24], which is part of the BBB of *Drosophila* [39,40], and the permeability of the BBB is impaired [41]. The BBB protects the central nervous system (CNS) from high concentrations of ions, especially potassium, elevated levels of which can disrupt electrical conductivity in the brain [42]. We also showed BBB permeability by injecting 10 kDa dextran into the abdomen of 5-day-old flies. Unlike WT flies, in which dextran was concentrated on the brain’s surface without penetrating into the brain, it was localized within the BBB and CNS in *sws*^1^ (Figure 7A,C).

The sheathing glia of the CNS envelop the entire neuropil, forming an internal barrier at the neuropil–cortex interface [43]. We analyzed this barrier by introducing dextran into the neuropil of the fly’s brain and analyzing its distribution using confocal microscopy. In WT, dextran was penetrated into the cerebral cortex of the neuropil in limited amounts. In the *sws*^1^ mutant, dextran filled the entire cavity of the cerebral ganglion cortex (Figure 7D,E; Supplementary Movies 1 and 2).

When analyzing the blood–eye barrier in WT flies, dextran was distributed at the retinal border. In *sws*^1^ flies, the dye was distributed within the retina, indicating disruption of the blood–eye barrier (Figure 7B). The structure of the retina was then visualized using phalloidin and Dlg-1 antibodies. Phalloidin is stained actin filaments enriched in the specialized photon-sensing domain known as rhabdomere, whose structure is violated in the *sws*^1^ mutant. Furthermore, visualization of the membrane-associated protein Dlg-1 in the retina showed an amendment in protein localization in the *sws*^1^ mutant (Figure 7B’). Therefore, it can be assumed that *sws* activity is required for SJ-mediated barrier integrity in multiple cells.

## 3. Discussion

Lipids are key components of membranes that determine their biophysical properties, in particular, their rigidity/curvature, lipid packing, fluidity and tension [44,45]. Phospholipid metabolism factors have been shown to be essential for individualization [46,47]. In mammals, the molecular composition of the sperm plasma membrane is known to change during maturation. A similar process is assumed to occur in *Drosophila* during individualization, when the molecular composition of sperm membranes is determined during IC migration. In this case, membranes can act as a depot for lipids [32].

In this study, we demonstrated a previously unknown function of *sws* in spermatogenesis at the stage of spermatid individualization. In the testes of *sws*^1^ flies, spermatid elongation occurs after meiosis, but not all spermatids are individualized. The ICs are formed and move caudally along the spermatid cyst. At the same time, the majority of spermatids do not encase themselves in their own membrane, indicating a disturbance in the membrane remodeling process that occurs simultaneously with IC movement [48]. The *sws*^1^ mutant also exhibits impaired spermatid coiling before their release into the ejaculatory duct. We also observed increased apoptosis during the final stages of spermatogenesis, which led to a reduction in the number of spermatozoa in the seminal vesicle. These data are consistent with the hypothesis that the coiling stage serves as a checkpoint for identifying abnormal spermatozoa following individualization [49,50].

The conducted transcriptomic analysis enabled a more comprehensive characterization of the testes of *sws*^1^ flies. Thus, among the downregulated DEGs, multiple mitochondrial function categories are significantly enriched, including the respiratory electron transport chain ATP synthesis coupled electron transport and mitochondrial membrane organization. Previously, we hypothesized that the observed reduction in sperm velocity might be linked to impaired energy supply. However, analysis revealed a decrease in testicular ATP levels in 15-day-old males, but not in 5-day-old males, even though the spermatozoa of the mutant males were less active. The results of a more sensitive method, RNA sequencing, point to earlier disruptions in mitochondrial function. This situation is exacerbated by reduced levels of alpha-tubulin, its acetylated form, and dynein, which leads to impaired flagellar motility and, consequently, a decline in overall sperm motility.

It should be noted that mitochondrial disorders were also shown by us earlier in the knockdown of *sws* in the nervous system of *Drosophila* [51].

Among upregulated DEGs, an enrichment of genes associated with septate junc-tions, including *bark*, *mesh*, *cora* and others, is also observed. Consequently, having hypothesized a disruption in the integrity of septate junctions in our flies, we analyzed the permeability of the somatic permeability barrier formed by these septate junctions. We found an impairment of barrier function during the late stages of spermatogenesis. However, not all of the levels of septate junction proteins we investigated were elevated. Conversely, the proteins Dlg-1, FasIII, and Nrg were weakly visualized using immunohistochemistry. In our view, these results are not contradictory; rather, they indicate the disruption of the composition and stability of the protein complexes forming the septate junction.

In this regard, it should be noted that our previous analysis of *sws* expression in male testes revealed a specific pattern. *sws* expression was absent in the apical region of the testis, where hub cells are localized, yet it was actively detected in the somatic cyst cells at a stage corresponding to the growth of mature cysts [25]. These cells, belonging to the squamous epithelium, become unusually thin and elongated during the growth of the dividing and differentiating germ cells they encapsulate [52]. They ultimately differentiate into head and tail cysts at the onset of individualization [53]. An important function of these cells is to create a physical barrier known as the somatic permeability barrier [54]. Electron microscopy studies have revealed the formation of septate junctions, similar to mammalian tight junctions, between two encapsulating somatic cells [55]. The main components of septate junctions are initially localized at the border of germ and somatic cyst cells and then condense between the two somatic cyst cells after meiosis [34].

Mature spermatozoa are highly polarized cells in *Drosophila*. Their polarization begins after the completion of meiosis, when the 64-cell spermatid cyst begins the differentiation process. Spermatid nuclei are grouped on one side of the cyst, while the flagellar axonemes grow on the other [56]. Subsequently, the elongating spermatid bundles must be correctly oriented relative to the apical–basal axis of the testes, which is important for the transfer of mature spermatozoa into the ejaculatory duct. Somatic cells of the cyst participate in this process: the head cyst cell encapsulates the clustered spermatid nuclei, while the tail cyst cell encloses the growing flagellar axonemes.

In turn, the somatic cyst cells are also polarized, with their apical membranes facing the germline and their basal membranes facing the outside of the cyst [52]. During the polarization process, extensive lipid rearrangement occurs, leading to differences in lipid composition between the apical and basolateral compartments of the plasma membrane [57,58]. To explain these differences, a mechanistic theory has been proposed involving lipid rafts, which are densely packed dynamic nanoscale structures that can fuse to form larger microdomains within cellular membranes, creating heterogeneity that leads to subcompartmentalization of both lipids and proteins in the membrane [59]. At the same time, recent data indicate that cell polarity may be mediated by more than just lipid raft function. The properties of the apical plasma membrane are mediated, at least in part, by its tightly packing lipidome [60]. It should be noted that *sws*^1^ males exhibit increased lipid droplet content, presumably in spermatid-containing cyst cells, further suggesting impaired lipid metabolism [25].

It was previously documented that one of the substrates of *sws* in vivo is phosphatidylcholine (PC), which is cleaved to glycerophosphocholine and two fatty acid molecules [16,60]. The *Drosophila* mutant of *sws*^1^ is characterized by an increased PC content and changes in the levels of other lipids [16], as well as mono- and polyunsaturated forms of PC and various forms of lyso-PC [60]. It is possible that abnormal lipid distribution resulting from the *sws*^1^ mutation leads to impaired membrane transport and membrane remodeling. It has been previously shown that increasing the concentration of lyso-PC causes membrane liquefaction [61]. Knockout of *PNPLA6* in retinal pigment epithelial cells in vitro and in vivo increased the levels of most molecular forms of phosphatidylethanolamine, phosphatidylserine, phosphatidylinositol, phosphatidylglycerol, and lysophosphatidylethanolamine compared with control cells, and it also impaired the availability of choline for phospholipid recycling [62]. Interestingly, among the upregulated DEGs in *sws*^1^ testes, the fatty acid biosynthetic process and triglyceride lipase activity are significantly enriched, which may indicate the development of a protective mechanism and compensatory response to impaired phospholipid metabolism. This hypothesis is supported by the accumulation of lipid droplets in *sws*^1^ mutant testes [25], presumably in the cyst cells, one of whose functions is to protect lipids from peroxidation [51,63].

The data we obtained indicate that the somatic cells of the cyst require normal expression of *sws* for their maintenance and functioning. Consequently, we hypothesize that decreased *sws* expression disrupts the function of cyst somatic cells in the last stages of spermatogenesis by critically altering phospholipid recycling in their plasma membranes, potentially altering the polarity of both the cells themselves and the spermatids. This, in turn, leads to disruption of membrane remodeling, destruction of septate junctions between these cells, and the formation of defective spermatozoa.

Expression of *sws* is detected in glia, spermatogenesis, the midgut, and Malpighian tubules [25,64]. Interestingly, *sws* function is required for the specific cell types. All of them are directly or indirectly involved in maintaining internal barriers formed by septate junctions in *Drosophila*. Thus, changes in *sws* expression in subperineural glial cells, which form the *Drosophila* blood–brain barrier [41], and *sws* knockdown in enterocytes [64], which form the intestinal barrier, manifested themselves as impaired barrier function and destruction of septate junction morphology. In this study, we demonstrated that the *sws*^1^ mutation also disrupts the somatic permeability barrier, the blood–eye barrier, and the intracerebral barrier formed by ensheathing glia. Taken together, these data suggest the universality of the sws mechanism of action across different cell types.

The similarity of the primary structure of the *sws* and mouse PNPLA6 proteins is 39% [65], and the similarity of their esterase domains is 61%, but this is sufficient for the functional interchangeability of the corresponding genes in experiments [16,64,66], which may indicate the similarity of the functions they perform. Thus, expression of human *PNPLA6* in enterocytes of *sws*^1^ mutants restored septate junctions and selective permeability of the intestinal barrier [64]. At the same time, mislocalization of zonula occludens/tight junction protein 1, a tight junction marker, is observed in *PNPLA6*-knockdown retinal pigment epithelial cells, suggesting insufficient formation of tight junctions [62].

## 4. Materials and Methods

### 4.1. Drosophila Stocks and Feeding

The flies were kept in standard semolina medium. The crosses were cultured at 25 °C (12 h night/12 h day).

The following fly stocks were used: *Canton S* (St. Petersburg State University fly collection, Saint-Petersburg, Russia), kindly donated by Elena Golubkova, hereinafter abbreviated as WT); the *sws*^1^ allele, described in [22] (kindly donated by Doris Kretzschmar); the *UAS-sws* line, described in [65]; the *eyaA3-GAL4* line, described in [66] (hereinafter abbreviated as *eya-GAL4*, kindly provided by Ludmila Olenina); and the *UAS-swsRNAi* line (#61338, Bloomington Drosophila Stock Center, Bloomington, IN, USA). To generate GFP expression in the subperineurial and ensheathing glia, the *UAS-CD8-GFP* line (#8746, Bloomington Drosophila Stock Center, Bloomington, IN, USA) and the glial drivers *NP2276-GAL4* (#112853, Drosophila Genetic Resource Center, Kyoto, Japan) and *NP6520-GAL4* (#105240, Drosophila Genetic Resource Center, Kyoto, Japan) were used.

### 4.2. Permeability Assay

To analyze the BBB and somatic permeability functionality, dextran 10 kDa conjugated Texas Red (Invitrogen by ThermoFisher Scientific, Waltham, MA, USA) was injected into the fly’s abdomen in a quantity of 0.5 µL. The flies were kept on a nutrient medium at 25 °C for 16 h. Next, the testis and brain were dissected in cold PBS with subsequent fixation of 4% paraformaldehyde for 30 min and placed in a mounting medium for fluorescent microscopy (Vectashield, Vector Laboratories, Newark, CA, USA).

To analyze the function of the ensheathing glia, dextran 10 kDa conjugated Texas Red was injected into the fly’s brain neuropil in a quantity of 0.05 µL [43]. The flies were maintained on a nutrient medium at 25 °C for 30 min. Next, the brain was dissected in cold PBS with subsequent fixation of 4% paraformaldehyde for 30 min and placed in a mounting medium for fluorescent microscopy.

Images of the brains were taken using a Leica TCS SP5 laser scanning confocal microscope (Leica, Wetzlar, Germany) with LAS AF software (version 4.13). For image processing, LAS X software (version 5.3.3) was used.

To analyze blood–eye barrier functionality, dextran 10 kDa conjugated Texas Red was injected into the fly’s abdomen in a quantity of 0.5 µL. The flies were kept on a nutrient medium at 25 °C for 2 h. Next, flies were fixed to a cover slip using double-sided tape, and their eyes were imaged using a Leica TCS SP5 fluorescence microscope (Leica, Wetzlar, Germany).

### 4.3. Immunohistochemical Staining and Microscopy

A total of 20 testes for each genotype were dissected in cold PBS and fixed in 4% paraformaldehyde for 30 min. Next, the samples were washed for 3 × 5 min in PBST solution (0.1% Triton and 1 × PBS) and incubated with primary antibodies diluted in blocking buffer (Visual Protein, BP01-1L; Taipei, Taiwan) at +4 °C overnight. After washing in PBS for 3 × 5 min, the samples were stained for 2 h at room temperature with a secondary antibody. The testes were washed for 3 × 15 min after immunohistochemical staining and placed in a mounting medium with Dapi (Abcam, Cambridge, UK). The following primary antibodies were used: mouse 4F3 (anti Dlg-1 (1:100)), mouse C566.9 (anti Cora (1:50)), mouse 7G10 (anti FasIII (1:50)), mouse BP 104 (anti Nrg (1:50)) (DSHB, Iowa City, IA, USA); rabbit Cleaved Caspase 3 (1:400; Cell Signaling Technology, Danvers, MA, USA); mouse AXO49 (1:500; Merck Millipore, Darmstadt, Germany). The following secondary antibodies were used: goat anti-mouse AlexaFluor488 (1:400; Abcam, Cambridge, UK), goat anti-mouse AlexaFluor633 (1:400; Abcam, Cambridge, UK) and goat anti-rabbit Alexa 594 IgG1 (1:400; Abcam, Cambridge, UK). For visualization of actin complexes, phalloidin labeled by iFluor488 was used (1:1000; Abcam, Cambridge, UK).

Images of the stained testes were taken using a Leica TCS SP5 laser scanning confocal microscope (Leica, Wetzlar, Germany) with LAS AF software (version 4.13). Image processing was performed using LAS X software (version 5.3.3).

### 4.4. Apoptosis Analysis

Testes were isolated in Schneider’s Drosophila Medium (Lonza, Bazel, Switzerland) without subsequent fixation. After that, the testes were incubated in YO-PRO^®^-1 iodide (ThermoFisher Scientific, Waltham, MA, USA) dissolved in PBS (working concentration: 3.1 mg/mL) for 20 min. Samples were then washed three times in PBS and placed in a mounting medium. Samples were analyzed after 15 min using a Leica TCS SP5 laser scanning confocal microscope with LAS AF software (version 4.13). Image processing was performed using LAS X software (version 5.3.3).

### 4.5. Electron Microscopy

Dissected testes were fixed in 2.5% glutaraldehyde in 0.1 M cacodylate buffer, pH 7.4, for 2 h and then postfixed in 1% osmium tetroxide in 0.1 M cacodylate buffer for 1 h. Treated tissues were stained with 1% uranyl acetate at 4 °C overnight. Following dehydration in ascending ethanol solutions, stained tissues were embedded in Epon. Ultrathin sections were prepared, stained with lead citrate, and examined using a JEOL 1400 transmission electron microscope (JEOL, Tokyo, Japan).

### 4.6. Library Preparation

Total RNA for RNA-seq libraries was isolated from Canton S and *sws*^1^ mutant testes (30 pairs per biological replicate) with the Quick-RNA MiniPrep kit. Ribosomal RNA (ZymoResearch, Irvine, CA, USA) was removed by hybridization of total RNA with biotinylated oligonucleotides complementary to different regions of rRNA followed by binding to Dynabeads™ MyOne™ Streptavidin C3 (Invitrogen by ThermoFisher Scientific, Waltham, MA, USA). RNA libraries were prepared utilizing the MGIEasy RNA Directional Library Prep Set (MGI, Shenzhen, China) according to the manufacturer’s protocol. Sequencing was conducted on the DNB-SEQ-G400 platform (MGI, Shenzhen, China) in paired-end reading mode, with a read length of 150 bp. Two biological replicates of each genotype were prepared.

### 4.7. RNA Sequencing Data Processing

Trimmomatic v0.40 was used to clean low-quality reads [67]. Hisat2 (version 2.2.2) [68] was used to map the filtered reads to the *Drosophila* reference genome [69]. htseq-count (version 2.0.3) [70] was used to count the number of reads that were mapped to the transcript. DESeq2 (version 1.52.0) [71] packages in R (version 4.2.0) were used to obtain lists of differentially expressed genes (DEGs). DEGs with a log2 fold change (log2FC) threshold greater than 0.3 were categorized as upregulated, while those with a log2FC below −0.3 were categorized as downregulated. The level at which statistically significant differences was considered was a *p*-value < 0.05.

### 4.8. Functional and Enrichment Analysis of DEGs

The packages “org.Dm.eg.db” (version 3.22.0) and “clusterProfiler” (version 4.18.4) [72] were used to analyze the enrichment of Gene Ontology pathways in biological processes, molecular functions and cellular components. REVIGO (version 1.8.2) was used to visualize biological processes, molecular functions and cellular components [73].

### 4.9. Validation of Transcriptome Analysis Results

For validation of transcriptome analysis results, wild-type and *sws*^1^ mutant testes were dissected in cold PBS. Total RNA was extracted from 30 pairs of testes per replicate for each genotype using Quick-RNA MiniPrep kit (ZymoResearch, Irvine, CA, USA). The M-MuLV–RH kit (Biolabmix, Moscow, Russia) was used for cDNA synthesis according to the manufacturer’s instructions. 5X qPCRmix-HS SYBR (Evrogen, Moscow, Russia) was used for the quantitative RT-PCR according to the manufacturer’s instructions. The reaction was carried out in triplicate using the CFX96 thermocycler (Bio-Rad, Hercules, CA, USA). The reaction conditions were: 40 cycles at 95 °C for 20 s, 59 °C for 15 s, and 72 °C for 20 s. Subsequently, melt-curve analysis was performed to verify the single-product presence in each reaction. The following primers were used (5′ to 3′) (Evrogen, Moscow, Russia): *protB* gene *AGAAGCACTGTGACTT-GAAGC* and *GACCTTGCATGCCATCCGGC*; *erasp* gene *TCTCATTACCCAGGGGTTTG* and *TCTCAGAGAACTTTCCATGCC*; *cg12376* gene *ACCATTCGTGTTTACTGTAATGAAC* and *ACGTTGGCAATTAGTGGAC*; *cyp4p3* gene *AAGATCACGCTGGTGTTTTTGAAC* and *ACAGTCCAGTTCTCAGAAATGG*; *tsf1* gene *GGAACCCATTTATCGCCTGTG* and *TAGGCGATGTACATGTCCTC*; *sdic4* gene *AGACACTGGTCTACACAAAGC* and *GCTCGTTGACCTCCTTCTTC*; *mesh* gene *ATCGACAACTCCCTCTACAC* and *TTGAATCCGACATATGCAGGAAC*; *bark* gene *ACACCAACCTCCAGAATTGTG* and *CAATGTCCAACTTCTGGAATTGG*.

As a reference gene, *RPL* was chosen, and the corresponding primers were the following (5′ to 3′): *ATGCTAAGCTGTCGCACAAATG* and *GTTCGATCCGTAAC-CGATGT*. Data analysis of qRT-PCR was performed with BioRad CFX Manager 3.1.

### 4.10. Determination of the Relative Content of Proteins

Just after the testes were dissected (20 testes per biological replicate, three replicates), they were frozen and stored at −80 °C. For protein isolation, frozen testes were homogenized in Laemmli buffer with the addition of a mixture of protease inhibitors (Calbiochem, San Diego, CA, USA). Then, samples were centrifuged and the total protein concentration in the supernatant was measured. Based on these measurements the same amount of protein was applied to each well of a polyacrylamide gel, and denaturing electrophoresis was performed (Bio-Rad Laboratories, Hercules, CA, USA) with subsequent transfer to a nitrocellulose membrane. The efficiency of the transfer was controlled by Ponceau staining; then, membranes were blocked in 4% milk (Skim milk powder #70166-500G; Sigma-Aldrich, Darmstadt, Germany) and stained with primary antibodies (for α-tubulin, 50 kDa—#ab52866, Abcam, Cambridge, UK; dilution: 1:10,000; for acetylated α-tubulin, 55 kDa—#sc-23950, Santa Cruz Biotechnology, Inc., Dallas, TX, USA; dilution: 1:1000; for dynein, 530 kDa—#14-9772-80, Invitrogen by Thermo Fisher Scientific, Waltham, MA, USA, eBioscienceTM; dilution: 5 µg/mL) and their corresponding HRP-conjugated secondary antibodies (anti-rabbit #7074S and anti-mouse #7076S; both obtained from Cell Signaling Technology, Danvers, MA, USA). Next, membranes were treated with substrates (SuperSignal™ West Femto Maximum Sensitivity Substrate; Thermo Scientific, Waltham, MA, USA), detected using the ChemiDoc XRS+ imaging system (Bio-Rad Laboratories, Hercules, CA, USA) and processed using Image Lab Software (Version 6.1, Bio-Rad Laboratories, Hercules, CA, USA).

### 4.11. Statistical Analysis

Statistical analysis was performed using KyPlot 5.0. software. All samples were tested for normality with the Shapiro–Wilk test. The Student’s *t*-test was used in case of a normal distribution. Data were presented as histograms (means ± 95% confidence intervals (CIs)). For other distribution types, the Mann–Whitney nonparametric test was applied. Data were presented in box plots.

## 5. Conclusions

Mutations in the *PNPLA6* and *sws* genes lead to the development of a variety of pathological phenotypes in *Drosophila* and humans, a complex form of SPG39 and a number of rare inherited diseases. In this study, we demonstrate the important role of the *Drosophila sws* gene in spermatogenesis and in the functioning of the biological barriers of the nervous system (the blood–brain barrier, the cortex–neuropil barrier and the blood–eye barrier) and the somatic permeability barrier during the late stages of spermatogenesis. Given the partial functional homology between PNPLA6 and *sws*, it can be assumed that they are involved in similar biological processes. We hope that this work will contribute to our understanding of the functions of *PNPLA6* and *sws*, particularly in maintaining the integrity and functioning of important internal barriers in the body in health and disease. This will contribute to a better understanding of the pathogenesis of these diseases and the development of therapeutic approaches for their treatment.

## Data Availability

The data presented in this study are available in the article and Supplementary Materials. The raw and processed data generated in this study have been deposited in the NCBI Gene Expression Omnibus (GEO) database and are accessible through GEO Series accession number GSE333384 (https://www.ncbi.nlm.nih.gov/geo/query/acc.cgi?acc=GSE333384, accessed on 27 May 2026).

## Supplementary Materials

The following supporting information can be downloaded at https://www.mdpi.com/article/10.3390/ijms27125486/s1.
