# Supplementary material for: Swiss cheese Is Essential for Maintaining Spermatogenesis and the Proper Functioning of Biological Barriers in Drosophila
Source: Int J Mol Sci. 2026 Jun 17;27(12):5486. doi: 10.3390/ijms27125486 (PMC13299251; doi:10.3390/ijms27125486)
Supplement: Supplementary file 1 [file ijms-27-05486-s001.zip › ijms-4302880-supplementary figure.pdf]

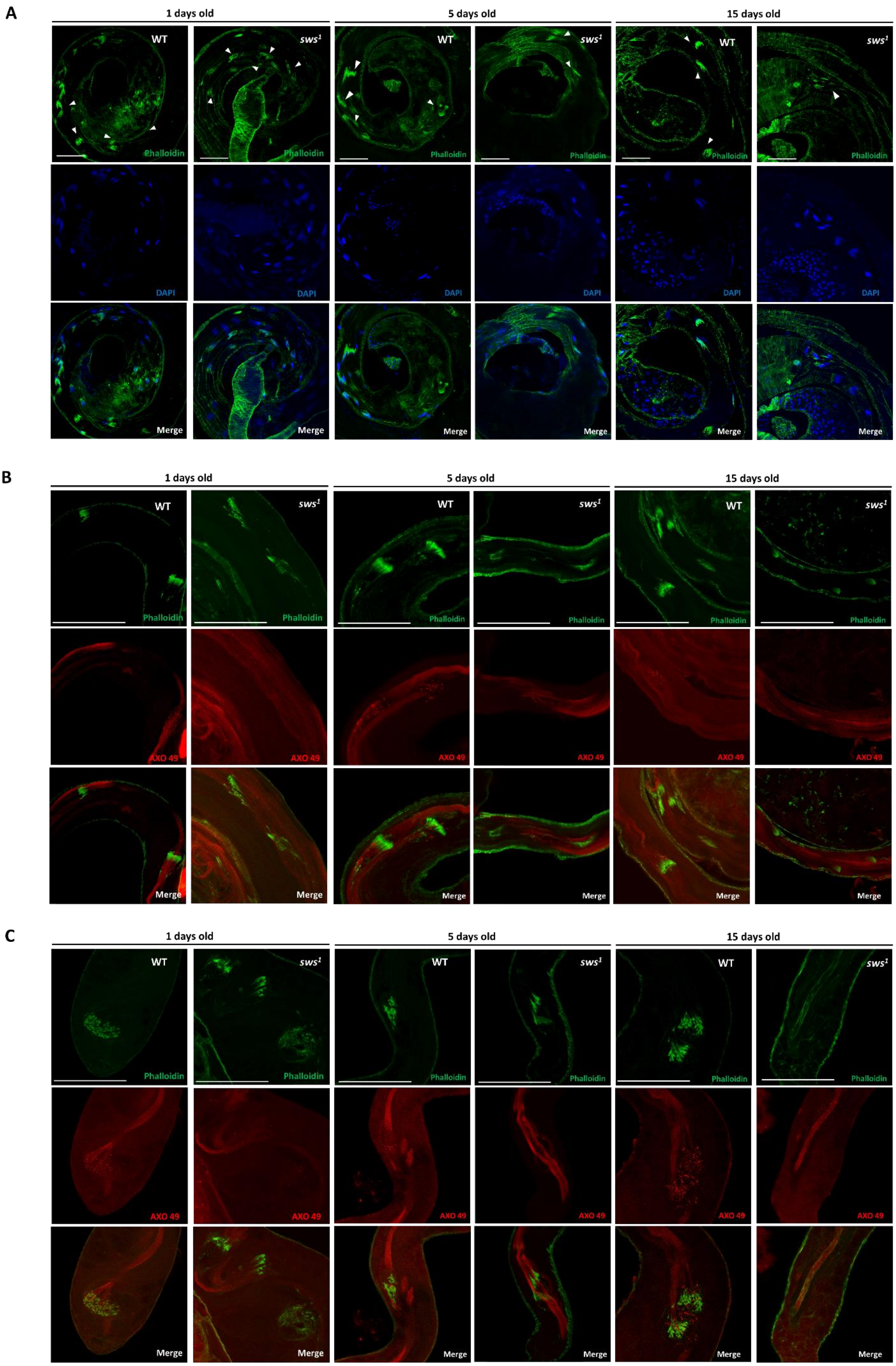
A
1 days old
5 days old
15 days old
WT
sws1
Phalloidin
DAPI
Merge
B
1 days old
5 days old
15 days old
WT
sws1
Phalloidin
AXO 49
Merge
C
1 days old
5 days old
15 days old
WT
sws1
Phalloidin
AXO 49
Merge

Fig. S1. The organization of individualization complexes. A – Individualization complexes in the terminal epithelium. White arrowheads show actin complexes. **B** – The individualization complexes move towards the apical end and waste bags are formed. **C** – Waste bags formed at the apical end of testis on individualization conclusion. Green – phalloidin, red - AXO49, blue - Dapi. Scale bar: 50 µm.

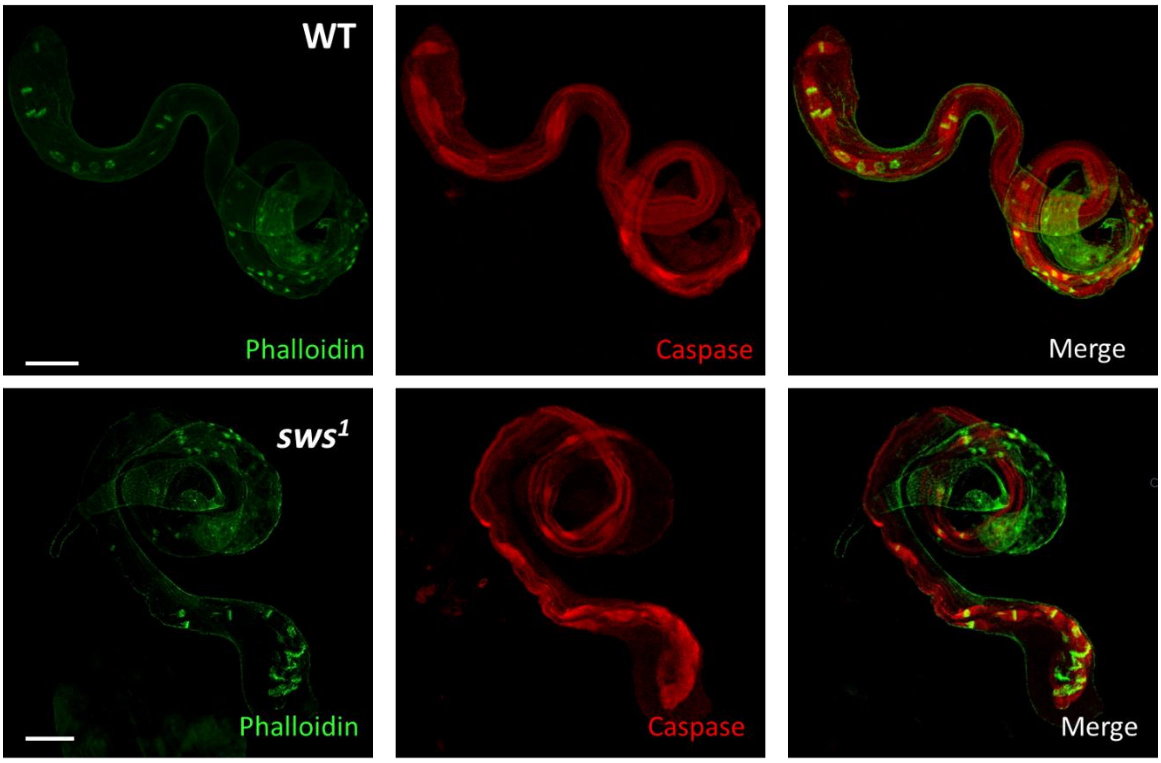

Fig. S2. Non-apoptotic caspase cascade in the cystic bulges. Red – Cleaved Caspase-3, green - phalloidin. Scale bar: 100 µm.

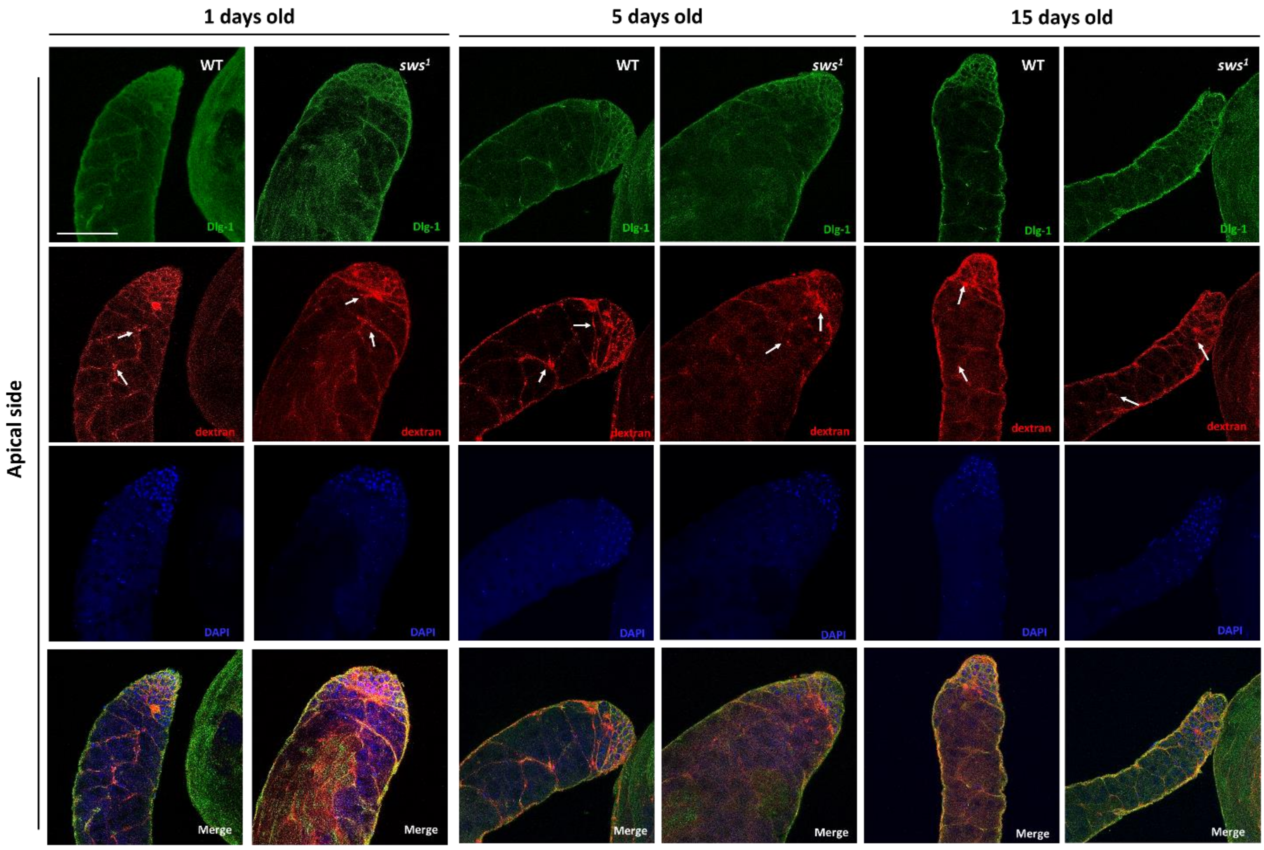

Fig S3. Somatic permeability barrier integrity on early phases of spermatogenesis. Permeability assay using 10kDa Dextran dye labeled Texas Red indicates that somatic permeability barrier is intact on early phases in wild type and mutant *sws*^{1} (White arrows). Green – DLG-1; red – dextran; blue – Dapi. Scale bar: 50 µm.

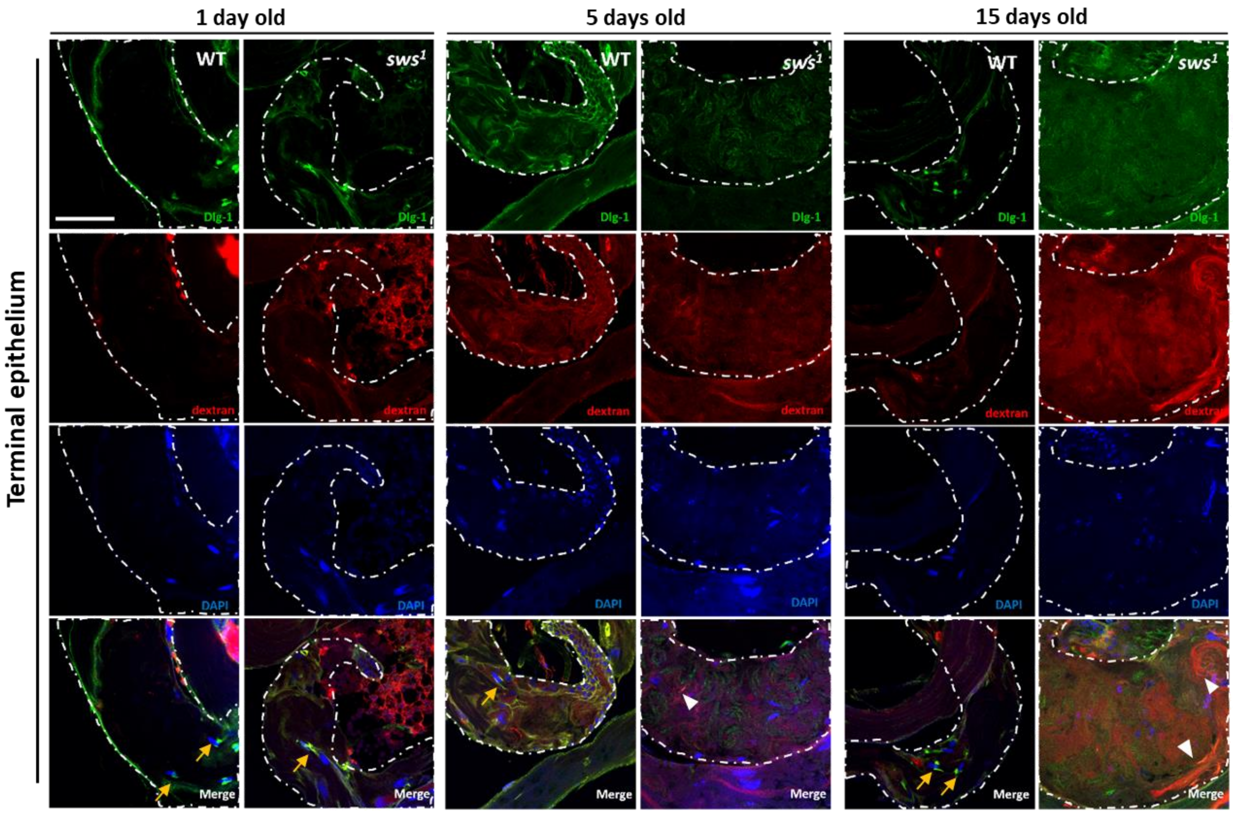

Fig.S4. Somatic permeability barrier integrity on late phases of spermatogenesis. The somatic permeability barrier becomes leaky in the *sws*^{1} mutant. Green – DLG-1; red – Dextran 10kDa; blue – Dapi. Yellow arrows show that Dlg-1 localizes caudal to the compact nuclei bundle of the mature spermatids during the late stages. White arrowheads show dextran localization in spermatids. Scale bar: 50 μm.
